# Genomic Prediction of Additive and Non-additive Effects Using Genetic Markers and Pedigrees

**DOI:** 10.1534/g3.119.201004

**Published:** 2019-07-01

**Authors:** Janeo Eustáquio de Almeida Filho, João Filipi Rodrigues Guimarães, Fabyano Fonsceca e Silva, Marcos Deon Vilela de Resende, Patricio Muñoz, Matias Kirst, Marcio Fernando Ribeiro de Resende Júnior

**Affiliations:** * Genetic Plant Improvenent Lab, Norte Fluminense “Darcy Ribeiro” State University, Campos dos Goytacazes RJ, 28013-602, Brazil,; † Futuragene Ltda, Itapetininga SP, 18207-780, Brazil,; ‡ Animal Science Department, Federal University of Viçosa, Viçosa, MG 36570-000, Brazil,; § EMBRAPA Forestry, Colombo, PR 83411-000, Brazil / Statistics Department, Federal University of Viçosa, Viçosa, MG 36570-000, Brazil,; ** Horticultural Sciences Department, University of Florida, Gainesville, FL 32611, and; †† University of Florida Genetics Institute, School of Forest Resources and Conservation, Gainesville, FL 32611

**Keywords:** Genotypic Value, Polygenic, Oligogenic, RKHS, BayesA, Genomic Prediction, GenPred, Shared Data Resources

## Abstract

The genetic merit of individuals can be estimated using models with dense markers and pedigree information. Early genomic models accounted only for additive effects. However, the prediction of non-additive effects is important for different forest breeding systems where the whole genotypic value can be captured through clonal propagation. In this study, we evaluated the integration of marker data with pedigree information, in models that included or ignored non-additive effects. We tested the models Reproducing Kernel Hilbert Spaces (RKHS) and BayesA, with additive and additive-dominance frameworks. Model performance was assessed for the traits tree height, diameter at breast height and rust resistance, measured in 923 pine individuals from a structured population of 71 full-sib families. We have also simulated a population with similar genetic properties and evaluated the performance of models for six simulated traits with distinct genetic architectures. Different cross validation strategies were evaluated, and highest accuracies were achieved using within family cross validation. The inclusion of pedigree information in genomic prediction models did not yield higher accuracies. The different RKHS models resulted in similar predictions accuracies, and RKHS and BayesA generated substantially better predictions than pedigree-only models. The additive-BayesA resulted in higher accuracies than RKHS for rust incidence and in simulated additive-oligogenic traits. For DBH, HT and additive-dominance polygenic traits, the RKHS- based models showed slightly higher accuracies than BayesA. Our results indicate that BayesA performs the best for traits with few genes with major effects, while RKHS based models can best predict genotypic effects for clonal selection of complex traits.

Pedigree and high-density DNA markers have been used to predict genetic merit of individuals in animal (Wiggans *et al.* 2011) and plant breeding (Resende *et al.* 2012a; Resende *et al.* 2012; Crossa *et al.* 2014). Initially, genomic prediction models were developed to select sires in animal breeding, accounting only for additive effects (Meuwissen *et al.* 2001). However, the prediction of dominance effects represents an important component of models designed for breeding program that focus on crossbred populations and/or hybrid production (Zeng *et al.* 2013; Nishio and Satoh 2014; Resende *et al.* 2017). In vegetatively-propagated species, non-additive effects are especially relevant because selection takes advantages of dominance effects. Hence, breeders can transfer whole-genotypic values of individuals to the next generation through clonal selection strategies.

Many statistical methods for genomic prediction have been proposed, which differ with regards to the assumed trait genetic architecture (Gianola 2013; de los Campos *et al.* 2013). For instance, BayesA consists of a whole-genome multiple regression (WGR) model where each marker regression coefficient assumes one specific variance (Meuwissen *et al.* 2001). Consequently, BayesA should provide a good fit for oligogenic traits where few genes explain a large proportion of the observed genetic variation (Meuwissen *et al.* 2001). However, prediction of non-additive effects introduces new SNP-covariates associated with these effects (Toro and Varona 2010), which may prohibitively increase the number of estimated parameters. The semi-parametric Reproducing Kernel Hilbert Space (RKHS) method has been proposed to account for non-additive effects, and is less computationally demanding than BayesA, especially when the number of individuals is substantially lower than the number of markers. RKHS combines features of non-parametric kernel regression with mixed-effect linear models (Gianola *et al.* 2006; Gianola and van Kaam 2008). RKHS is equivalent to the animal model in BLUP (de los Campos *et al.* 2009a), but uses kernel matrices that are different than the numerator relationship matrix (Morota *et al.* 2013; Tusell *et al.* 2014; Morota and Gianola 2014). In fact, the numerator relationship matrix is a special case of RKHS matrices.

Empirical results showed that the inclusion of dominance in BayesA improved the prediction accuracy in complex real and simulated traits in a pine breeding context. The additive-dominance BayesA was also superior to other Bayesian models for oligogenic traits (de Almeida Filho *et al.* 2016). In addition, reports that analyzed complex traits in other forest species showed improvement of accuracy in genomic selection models with inclusion of pedigree information in additive-only models (Beaulieu *et al.* 2014a, b). Thus, it is hypothesized that inclusion of pedigree information in additive-dominance BayesA can also increase prediction accuracy in structured breeding populations. The same hypothesis can be considered for alternative methods to BayesA, such as RKHS, which may also provide higher prediction accuracies when combined with pedigree data (Crossa *et al.* 2010, 2013).

To date, few studies have compared the prediction of non-additive effects from models using only genetic marker data, with models that integrate genetic markers and pedigree information. Moreover, the ability of RKHS models to predict whole genotypic values has not been assessed in the context of forest tree breeding. This study had as objectives: 1) evaluate the impact of including pedigree information in genomic prediction models applied to forest tree breeding, 2) evaluate RKHS and compare it with BayesA when it includes non-additive effects or not and 3) contrast different cross-validation strategies in the context of a structured breeding population. We applied and evaluated these methods to traits with distinct genetic architecture measured in a loblolly pine breeding population, and to simulated oligogenic and polygenic traits with different levels of dominance.

## Material and Methods

### Loblolly pine population and phenotypes

In this study, we used the traits tree height (HT), diameter at breast height (DBH) and two measures of fusiform rust infection: presence or absence of rust (RFbin) and gall volume (RFgall). These traits were measured in the loblolly pine (*Pinus taeda)* CCLONES breeding population, as previously described (de Almeida Filho *et al.* 2016). The traits HT and DBH are polygenic (Resende *et al.* 2012b), and HT also shows a significant non-additive genetic contribution (Muñoz *et al.* 2014). In contrast, rust resistance is thought to be controlled by fewer genes, including loci of large effect (Resende *et al.* 2012b; Quesada *et al.* 2014). The CCLONES population where these traits were measured was created from 42 founders, which were crossed to create 71 full-sib families, with an average of 13 individuals per family (SD = 5). In total, 923 individuals were genotyped for 7,216 single nucleotide polymorphic markers (SNP) as previously described (de Almeida Filho *et al.* 2016). From the total set, all 4,722 polymorphic loci were used in this study. Traits HT and DBH were measured in a field trial established in Nassau under alpha lattice design (Florida, USA), when the plants were six years old, in eight clonal replicates (Baltunis *et al.* 2007). Rust resistance traits (RFbin and RFgall) were measured in greenhouse, under randomized complete block design, as previously described (Kayihan *et al.* 2005, 2010). The phenotypes for these traits were adjusted with the following linear model:

$$y_{ijk}=\mu +b_{k}\left(r_{j}\right)+r_{j}+g_{i}+e_{ijk}$$

Where $y_{ijk}$ is phenotype of the i^th^ clone evaluated in the j^th^ repetition and the k^th^ incomplete block, μ is the intercept,$\;b_{k}\left(r_{j}\right)$ is the random effect of k^th^ incomplete block nested in the j^th^ repetition $b_{k}\left(r_{j}\right)∼N(0,\sigma_{b}^{2})$, $r_{j}$ is the fixed effect of the j^th^ repetition and $g_{i}$ is the effect of the i^th^ clone (considered as fixed to estimate the least-square means or adjusted means) and $e_{ijk}$ is the error of observation ijk $e_{ijk}∼N(0,\sigma^{2})$. This model was used for analysis of DBH and HT. For rust resistance, the incomplete block term was excluded. The analysis of variance of these traits is described in Table S1.

### Simulated population and phenotypes

We also analyzed six traits with different genetic architecture, in a simulated population described previously (de Almeida Filho *et al.* 2016). This analysis included traits with oligogenic and polygenic inheritance, and three levels of dominance (none, median and high). The simulated population was created following a standard forest breeding program model, in two steps. First, a base population with 1,000 individuals was created by randomly sampling 2,000 haplotypes from a population with effective size of 10,000, generated by 1,000 generations of a neutral coalescence model, with mutation rate 2.5 × 10^−8^ per generation (Willyard *et al.* 2007). Next, the breeding population was created by phenotypic selection of 100 individuals from the base population. These selected individuals were randomly mated to create 1,000 individuals to be used in the first breeding cycle. From the 1,000 individuals of the first breeding cycle, 42 were phenotypically selected and crossed following the same mating design used in the loblolly pine CCLONES population (Baltunis *et al.* 2007). Ten independent replicates were carried out for each simulated trait.

The simulated genome had 12 chromosomes with 100 cM. A set of 10,000 bi-allelic loci (*e.g.*, SNP) were used in developing prediction models, and 30 or 1,000 QTL were simulated for oligogenic and polygenic traits, respectively. All traits had narrow sense heritability of 0.25. Three levels of dominance were evaluated: *d^2^*: 0, 0.1 and 0.2, where *d^2^* is the proportion of phenotypic variance explained by dominance deviation - $d^{2}=V_{d}/V_{p}$; $V_{d}$ and $V_{p}$ are dominance deviation and phenotypic variances respectively (Falconer and Mackay 1996). The additive effect of a gene (a) was defined as half of the difference between the alternative homozygotes, and the dominance effect (d) was estimated by the difference between the heterozygote and the mean of the homozygotes. The distribution used for a in oligogenic traits were gamma (rate = 1.66, shape = 0.4) with sign (positive or negative) sampled with equal probability (Meuwissen *et al.* 2001). For polygenic traits, a was simulated with standard normal distribution (mean = 0, sd = 1) (Hickey and Gorjanc 2012). When dominance was present, it was simulated by: $d_{i}=a_{i}\times \tau_{i}$, where $\tau_{i}$ was sampled from a normal distribution with mean zero and standard deviation 1 and 2 for traits with medium- and high-dominance-levels, respectively. To achieve the targeted values of *d^2^*, only samples that provided *d^2^* between 0.09 and 0.11 for medium-dominance traits and between 0.19 and 0.21 for high-dominance traits were kept. The simulated populations showed very similar allele frequencies, when compared across scenarios. Hence, *d^2^* was mostly defined by a larger or smaller sampled value of $\tau_{i}$.

### Statistical methods

We evaluated models that consider just SNP or pedigree information, and models that combined both. The DNA marker (SNP) component was fitted with the following methods: a) semi parametric Reproducing Kernel Hilbert Space models (RKHS), using different kernels (Ka and Ka-Kd), and b) BayesA considering additive and additive-dominant effects. BayesA was the choice of WGR because it was previously shown to generate better predictions than the Bayesian Lasso and Bayesian Ridge Regression, and to have similar accuracies as BayesB, for oligogenic traits (de Almeida Filho *et al.* 2016). The full base model can be represented by:

$$y_{j}^{*}=\mu +g_{j}+u_{j}+\delta_{j}+e_{j}$$

Where $y_{j}^{*}$ is the phenotype (adjusted clonal mean in real traits) of individual *j*; µ is the intercept; $g_{j}$ is the genotypic value for each j*^th^* individual, estimated from SNP data — this term changed among methods adopted, as can be seen bellow; *u_j_* is the additive polygenic effect (when included) of individual j; *δ_j_* is the dominant polygenic effect (when included) of individual j; $e_{j}$ is the error term. The joint data distribution, the prior distribution for the constant *μ*, and the prior distributions for the vectors g, *u* and *δ* (containing information of all evaluated individuals) are given by:

$$y_{j}^{*}|\mu ,g_{j},u_{j},\delta_{j},\sigma_{e}^{2}∼IID\;N(\mu +g_{j}+u_{j}+\delta_{j},\sigma_{e}^{2});$$

$$\mu ∼N(0,10^{6});$$

$$u|A\sigma_{u}^{2}∼N(0,A\sigma_{u}^{2});\;\mathrm{and}$$

$$\delta |D\sigma_{\delta }^{2}∼N(0,D\sigma_{\delta }^{2}).$$

Where A and D are the additive relationship matrix and dominance relationship matrix, respectively, calculated using standard methods (Henderson 1984; Lynch and Walsh 1998; Mrode 2014). In the pedigree model, the $g_{j}$ term was absent and u and δ are the breeding values and dominance deviation vectors, respectively.

#### BayesA:

The BayesA model adopted in this study used the changes in the formulation proposed by Gianola *et al.* (2009), modified from the original version (Meuwissen *et al.* 2001) to estimate the shape parameter of the inverted chi-square ($\chi^{-2}$). This modification is expected to reduce the influence of the hyperparameter and consequently improve the bayesian learn. The full BayesA model included additive and dominant effects from SNP and pedigree. This model can be represented by:

$$y_{j}^{*}=\mu +\sum_{i=1}^{k}\left(x_{ij}a_{i}+w_{ij}d_{i}\right)+u_{j}+\delta_{j}+e_{j}$$

Where $x_{ij}$ and $w_{ij}$ are the functions of SNP *i* in individual *j*, for genotypes AA, Aa and aa. $x_{ij}$ is composed of values 1 (AA), 0 (Aa) and -1 (aa) and $w_{ij}$ of 0 (AA), 1 (Aa) and 0 (aa). $a_{i}$ and *d_i_* are the additive and dominance effect of marker *i*, respectively. The dominance effect was fitted only in the additive-dominance model. The priors used in linear regression coefficients for additive-dominance and additive models are described below:

$a_{i}|\sigma_{a_{i}}^{2}∼N(0,\sigma_{\alpha_{i}}^{2})$;$\;\sigma_{a_{i}}^{2}|\nu_{a},S_{\alpha }∼\chi^{-2}(\nu_{a},S_{\alpha })$;$S_{a}|s_{a},r_{a}∼G(s_{a},r_{a})$; $d_{i}|\sigma_{d_{i}}^{2}∼N(0,\sigma_{d_{i}}^{2})$; $\;\sigma_{d_{i}}^{2}|\nu_{d},S_{d}∼\chi^{-2}(\nu_{d},S_{d})$; $\;S_{d}|s_{d},r_{d}∼G(s_{d},r_{d})$. Where $\chi^{-2}$ and *G* represent the scaled inverse chi square and Gamma distributions, respectively.

#### RKHS Kernel averaging model:

From the full base model represented above, the term *g* in RKHS represents the whole genotypic values explained by the markers, including additive and non-additive effects such as dominance and gene interactions (Gianola *et al.* 2006; Gianola and van Kaam 2008). Here, g was modeled in two forms referred to as RKHS Ka and RKHS Ka-Kd.

##### RKHS-Ka:

In the RKHS-Ka, the whole genotypic effect (g) was explained by 3 SNP functions:

$$g=\sum_{r=1}^{3}g_{r}$$

where

$$g_{r}|K_{a}{}_{r}\sigma_{g_{r}}^{2}∼N(0,K_{a_{r}}\sigma_{g_{r}}^{2})$$

$$\sigma_{g_{r}}^{2}|\nu_{g},S_{g}∼\chi^{-2}(\nu_{g},S_{g})$$

$$K_{a_{r}}=\text{exp}(-\phi_{a_{r}}D_{a}^{2})$$

$D_{a}^{2}$ is the square of the Euclidean distance matrix among the individuals using the SNP additive incidence matrix **X** that takes values -1, 0 and 1. The $\phi_{a}$ is a bandwidth parameter that controls for the relationship measure between individuals j and j’, for a given distance (squared Euclidean in this case). Large positive values of bandwidth result in the relationship of j and j’ being close (or equal) to 0. Small positive values result in the relationship of j and j’ being close (or equal) to 1. The kernel averaging method (De los Campos *et al.* 2010) was used to determinate the bandwidth components in this study. In this approach, each SNP function g is replaced for two or more SNP functions with the same distance (squared Euclidean in this case), but with different bandwidth values — in kernel averaging these bandwidths are not regular parameters, they are constants. The bandwidth values $(\phi_{a}{}_{r}\;$) used in $g_{1}$, $g_{2},$ and $g_{3}$ are 5/h, 1/h and 0.2/h respectively, where *h* is 5^th^ percentile of $D_{a}^{2}$ leading to local, intermediate and global kernels, respectively (González-Camacho *et al.* 2012; Tusell *et al.* 2014).

##### RKHS-Ka-Kd:

In addition to the information contained in the **X** matrix described above, RKHS-Ka-Kd also includes **W**, that is the SNP incidence matrix for dominance effects in additive-dominance Bayes A. The g in this case for each SNP matrix (X and W) used three SNP functions:

$$g=\sum_{r=1}^{3}(g_{a_{r}}+g_{d_{r}})$$

The $g_{a}{}_{r}$ consists of the same SNP functions used in RKHS-Ka, and for $g_{d_{r}}$ we assumed:

$$g_{d}{}_{r}|K_{d_{r}}\sigma_{gd_{r}}^{2}∼N(0,K_{d_{r}}\sigma_{gd_{r}}^{2}\;)$$

$$\sigma_{gd_{r}}^{2}|\nu_{g_{d}},S_{g_{d}}∼\chi^{-2}(\nu_{g_{d}},S_{g_{d}})$$

$$K_{d_{r}}=\text{exp}(-\phi_{d_{r}}D_{d}^{2})$$

$D_{d}^{2}$ is the square of the Euclidean distance matrix using the dominance SNP matrix **W** that takes values 1 for heterozygote and 0 for both homozygotes, as described in the additive-dominance-BayesA model. The same bandwidth values used in RKHS-KA were used for $g_{a_{r}}$ in RKHS-Ka-Kd, and for $g_{d_{1}}$, $g_{d_{2}}$ and $g_{d_{3}}$, the bandwidths values ($\phi_{d_{r}}$) were 5/h_d_, 1/h_d_ and 0.2/h_d_ respectively, where h_d_ is 5^th^ percentile of $D_{d}^{2}$, similar to Morota *et al.* (2014). In both RKHS models (Ka and Ka-Kd) the whole genotypic value was predicted but cannot be separated into the components: breeding value, dominance deviation and epistasis. The summary of models compared are available in Table S2.

### Models validation

The prediction accuracies were calculated using 10-fold cross-validation (Resende *et al.* 2012b; de Almeida Filho *et al.* 2016). In order to infer the impact of the training population (TP) on the prediction accuracy, the TP was defined following three different schemes: a) prediction across families –individuals from a group of families were used to fit the model and the genetic potential was predicted in individuals from different families; b) prediction within families – the genetic merit of was predicted in individuals belonging to the same families included in the TP; c) random sample of individuals for TP. These validation approaches were also applied to each of the ten replicates of the simulated data. For data collected from the CCLONES population, the 10-fold based on random sample process was applied 10 times with independent groups of individuals in each fold. Prediction of accuracies and regression coefficients of parametric values on validation data were estimated for each of the 10 folds. A graphical representation of the 10-folds cross-validation schemes used in this study is available in Figure S1.

### Breeding values and dominance deviation

The expected breeding value (EBV) and the expected dominance deviation (EDD) were estimated as described below:

$$E\hat{B}V_{j}=\underset{i}{\sum }\left[I\left(x_{ij}=1\right)2q_{i}+I\left(x_{ij}=0\right)\left(q_{i}-p_{i}\right)-I(x_{ij}=-1)2p_{i}\right]{\hat{\alpha }}_{i}+{\hat{u}}_{j}$$

and

$$E\hat{D}D_{j}=\underset{i}{\sum }\left[-I\left(x_{ij}=1\right)2q_{i}^{2}+I\left(x_{ij}=0\right)2p_{i}q_{i}-I\left(x_{ij}=-1\right)2p_{i}^{2}\right]{\hat{d}}_{i}+{\hat{\delta }}_{j}$$

Where *p_i_* is allele frequency of allele A of SNP *i*, *q_i_ = 1-p_i_*, ${\hat{\alpha }}_{i}$ is the average effect of substitution, ${\hat{\alpha }}_{i}=\;{\hat{a}}_{i}+{\hat{d}}_{i}(q_{i}-p_{i})$, and I is an indicator function of SNPs; ${\hat{u}}_{j}$ and ${\hat{\delta }}_{j}$ are terms from additive and dominant polygenic effects, respectively. The whole genotypic value is the sum of $E\hat{B}V_{j}$ and$\;E\hat{D}D_{j}$. In RKHS-based models, the whole genotypic value is predicted, and therefore the partitions between $E\hat{B}V_{j}$ and$\;E\hat{D}D_{j}$ cannot be estimated.

### Variance components

The variance components from WGR are extensions of estimators previously reported (Zeng *et al.* 2013; Ertl *et al.* 2014) that assume absence of epistasis, linkage equilibrium among markers and Hardy-Weinberg equilibrium (Gianola *et al.* 2009). The general estimator of additive variance (*V_A_*) and the variance due to dominance deviation (*V_D_*) are:

$${\hat{V}}_{A}=2\underset{i}{\sum }p_{i}q_{i}\left[{\hat{\sigma }}_{a_{i}}^{2}+{\left(q_{i}-p_{i}\right)}^{2}{\hat{\sigma }}_{d_{i}}^{2}\right]+{\hat{\sigma }}_{u}^{2}$$

and

$${\hat{V}}_{D}=4\underset{i}{\sum }{\left(p_{i}q_{i}\right)}^{2}{\hat{\sigma }}_{d_{i}}^{2}+{\hat{\sigma }}_{\delta }^{2}$$

The components ${\hat{\sigma }}_{u}^{2}$ and ${\hat{\sigma }}_{\delta }^{2}$ in each model above are associated with the polygenic effect. The remaining equation of ${\hat{V}}_{A}$ and ${\hat{V}}_{D}$ are due to marker effects. The whole genotypic variance is the sum of additive and dominance variance. The *h^2^*, *d^2^* and *H^2^* are the proportion of additive, dominance and genotypic variance in the phenotypic variance. In RKHS models the genetic variance estimated by markers is the whole genotypic variance. All models were fitted with the R package BLGR (de los Campos and Perez 2014), using 100,000 iterations, burn-in of 20,000, thinning of 3 and default hyperparameters previously described (Pérez and de los Campos 2014).

### Data availability

All phenotypic and genotypic data utilized in this study have been previously published as a standard data set for development of genomic prediction methods (Resende *et al.* 2012b). Simulated data available from the Dryad Digital Repository: http://dx.doi.org/10.5061/dryad.3126v. Supplemental material available at FigShare: https://doi.org/10.25387/g3.8379059.

## Results

### The prediction within families was more accurate than prediction across families

**Figure 1:**
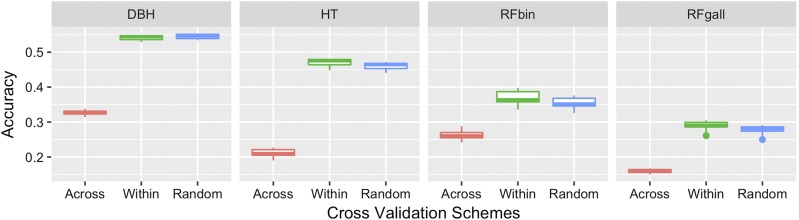
Accuracy distribution of all genomic prediction models fitted for tree height (HT), diameter at breast height (DBH) and two measures of fusiform rust infection: presence or absence of rust (RFbin) and gall volume (RFgall). These results were achieved from three different 10-fold cross validation orientations: a) Across families: Each fold is a group of distinct families; b) Within families: The folds were grouped inside families; and c) Random sample: Each fold is a group of distinct individuals random sampled ignoring family information.

**Figure 2:**
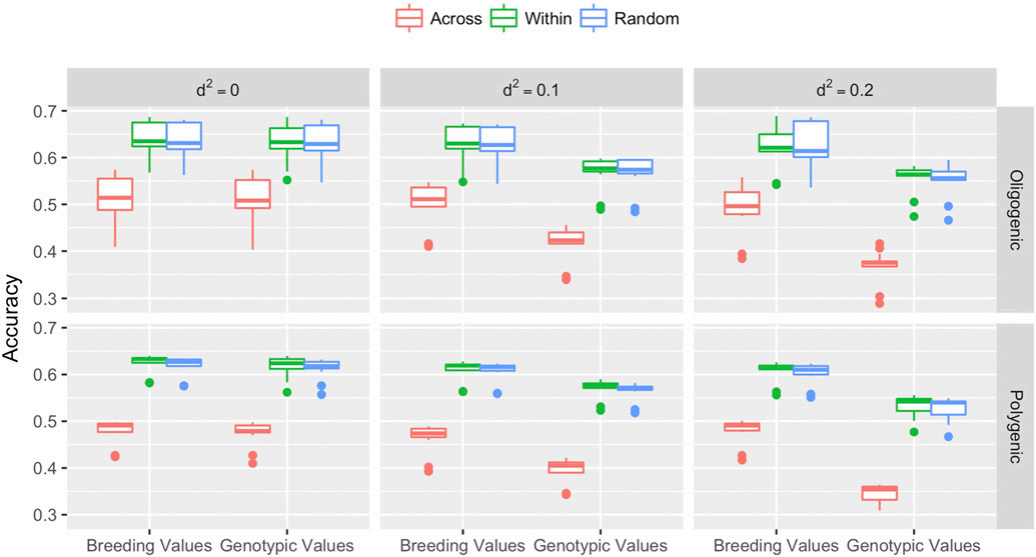
Accuracy average of prediction of breeding values and genotypic values for all genomic prediction models fitted in six simulated traits: Oligogenic and Polygenic with three degrees of dominance (d^2^ = 0; d^2^ = 0.1 and d^2^ = 0.2). These results were achieved from three different 10-fold cross validation orientations: a) Across families: Each fold is a group of distinct families; b) Within families: The folds were grouped inside families; and c) Random sample: Each fold is a group of distinct individuals random sampled ignoring family information.

To infer the impact of population structure and the choice of training population (TP) in the estimates of prediction accuracy, we carried the cross-validation across families, within families and at random. For all models and traits, the prediction accuracies were lowest when the predicted individuals came from families not included in the TP (across family cross-validation). The reductions in accuracy ranged from ∼15–59% in both real and simulated data (Figure 1-2). Conversely, the within-family cross validation resulted in highest accuracy for the majority of cases. In CCLONES, the superiority of the within-family TP ranged from 1.5–2.4%, 2.7–6.8% and 3.6–5.6% for HT, RFbin and RFgall, respectively (Figure 1). In simulated traits, the within-family cross validation was slightly superior (0.15–3.3%) than the random sample TP in ∼85% and ∼80% of cases for prediction of breeding values and whole genotypic values respectively (Figure 2). There model comparison, however, was very similar across TP schemes, and no interaction between models and TP was observed (data not shown). Hence, the following model comparisons and general conclusions, were performed using random-sample cross-validation.

### Pedigree information in model prediction

The use of pedigree information has been shown previously to improve the accuracy of genotypic predictions (Crossa *et al.* 2014). Here, we evaluated if this gain in accuracy is also observed in our populations, based on correlation using parametric genetic values from simulated data, and phenotypic values from real data. Initially we compared the prediction accuracy of models based only on pedigree information against models with markers-only data (Table 1). Overall, models based only on pedigree data had modestly lower accuracy than those using marker information for traits measured in the pine CCLONES population. The improvement in accuracy for breeding and genotypic values prediction was much more apparent when markers were used in the simulated population, compared to when only pedigree data were utilized. This improvement was particularly pronounced for simulated oligogenic traits (Table 2). Next, we extended the comparison of accuracies to include models that combined both pedigree and marker information. We observed that in only few instances the expanded models had higher accuracies, and that in general the accuracies were comparable to those with marker only in the real and simulated data (Table 1-2). One of such cases was the accuracy in predicting dominance deviation in the simulated traits (Table 2).

**Table 1:** Average of accuracies for prediction of phenotypic values for all models based in pedigree-only, in markers-only and models combining pedigree and markers information

Models	DBH	HT	RFbin	RFgall
Pedigree	0.536	0.450	0.331	0.255
Markers	0.545	0.459	0.361	0.288
Markers + Pedigree	0.548	0.465	0.356	0.279

Only-Markers models: Additive-, Additive-dominance-BayesA, RKHS Ka and RKHS Ka-Kd; Only-Pedigree models: Additive-, Additive-dominance-Pedigree; Markers + Pedigree are the models id: 2,4,5,7,8,10,11 (Table S2).

**Table 2:** Average of accuracies of breeding values, dominance deviation, genotypic values and phenotypic values prediction for all models based in pedigree-only, in markers-only and models combining pedigree and markers information

Accuracy	Models	d^2^ = 0		d^2^ = 0.1		d^2^ = 0.2	
		Olig	Poly	Olig	Poly	Olig	Poly
Breeding Value	Pedigree	0.564	0.576	0.545	0.560	0.538	0.554
	Markers	0.653	0.627	0.645	0.618	0.645	0.613
	Markers + Pedigree	0.646	0.626	0.639	0.615	0.638	0.610
Dominance Deviation	Pedigree	—	—	0.179	0.202	0.271	0.259
	Markers	—	—	0.175	0.169	0.273	0.243
	Markers + Pedigree	—	—	0.186	0.185	0.284	0.258
Genotypic Value	Pedigree	0.556	0.567	0.488	0.521	0.481	0.479
	Markers	0.652	0.626	0.586	0.575	0.569	0.537
	Markers + Pedigree	0.638	0.619	0.578	0.571	0.566	0.536
Phenotypic Value	Pedigree	0.251	0.259	0.284	0.306	0.313	0.335
	Markers	0.297	0.286	0.338	0.331	0.378	0.373
	Markers + Pedigree	0.290	0.282	0.335	0.331	0.373	0.373

Only-Markers models: Additive-, Additive-dominance-BayesA, RKHS Ka and RKHS Ka-Kd; Only-Pedigree models: Additive-, Additive-dominance-Pedigree; Markers + Pedigree are the models id: 2,4,5,7,8,10,11 (Table S2).

### Genotypic predictive model accuracy depends on trait architecture

**Figure 3:**
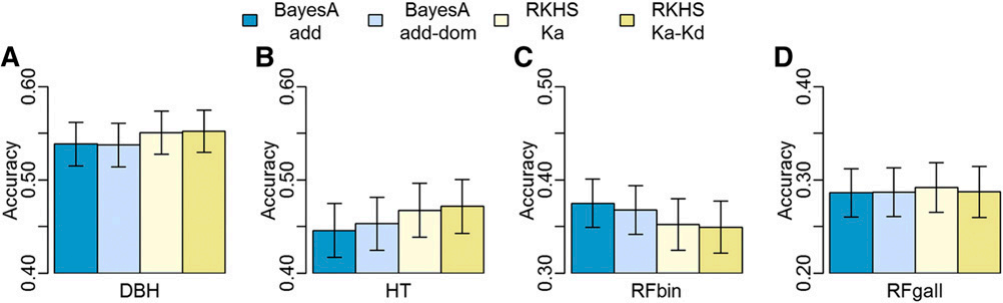
Average of phenotypic prediction accuracies and standard error for four markers-only models: additive- and additive-dominance-BayesA, RKHS-Ka and RKHS Ka-Kd for diameter at breast height (DBH), height (HT) and Rust resistance evaluated as gall volume (RFgall) and presence or absence (RFbin) in loblolly pine. The standard errors (s.e.=s.d.(x)/sqrt(10)) were calculated for each ten-fold procedure. The error bars are the averages of s.e. across the ten independent cross validations.

**Figure 4:**
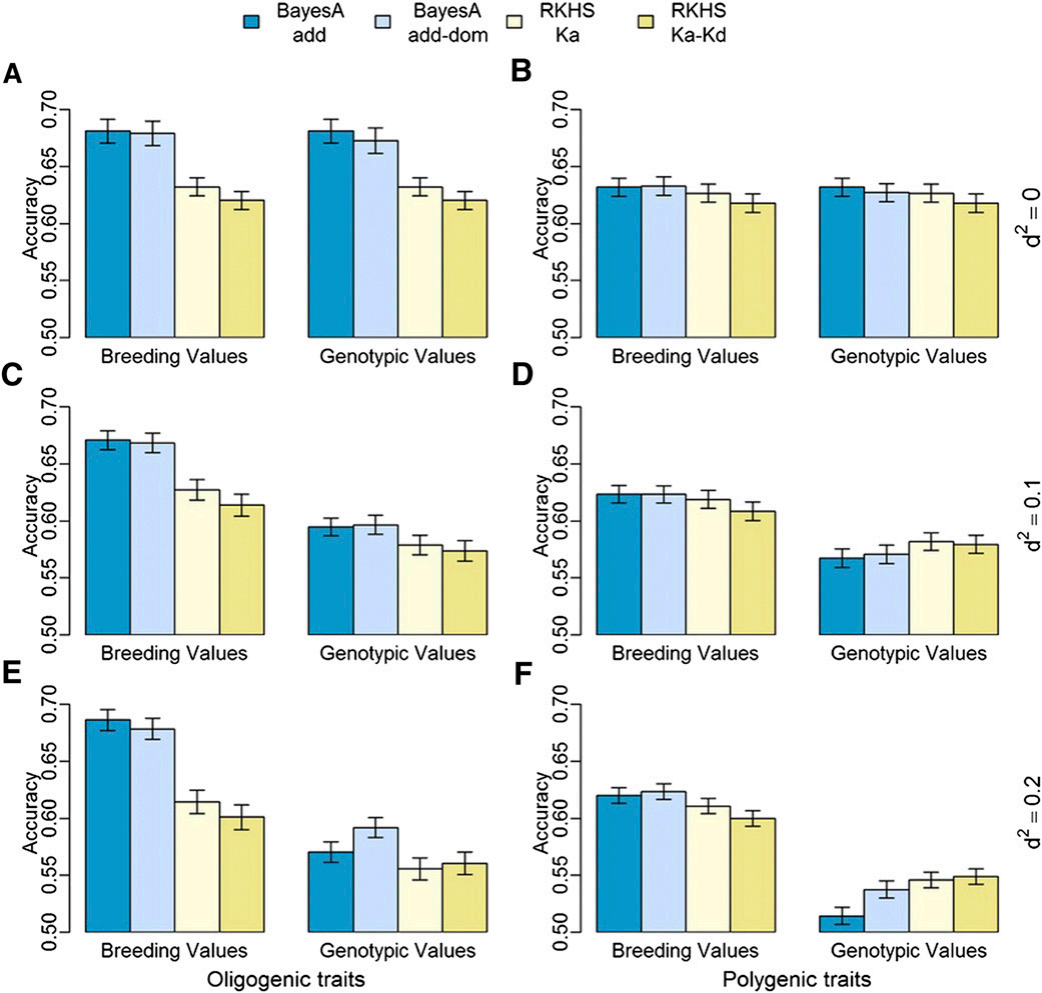
Accuracy average of prediction of breeding values and genotypic values for four marker-only models: additive-, additive-dominance-BayesA, RKHS-Ka and RKHS Ka-Kd for six simulated traits: Oligogenic and Poligenic with three degrees of dominance (d^2^ = 0; d^2^ = 0.1 and d^2^ = 0.2). Error bars are standard error (s.e.) considering the 100 independent samples used to calculate the mean (s.e.=s.d.(x)/sqrt(100)).

The two genomic prediction methods tested in this study differ with respect to the assumptions regarding the genetic architecture of the trait being predicted. BayesA is a linear regression model that assumes that each marker has different variance. Consequently, some markers can explain the effect of major loci, such as in oligogenic traits (*e.g.*, rust resistance). On the other hand, RKHS is a semi-parametric model that assumes that all markers with the same MAF contribute equally to the relationship among individuals. Thus, this assumption has closer resemblance with polygenic trait regulation. For phenotypic predictions, RKHS had higher accuracy for DBH and HT (Figure 3) when compared to BayesA. Similar results were observed in the polygenic traits with presence of dominance effects (*d^2^* = 0.1 and *d^2^* = 0.2). In both cases, the RKHS method resulted in higher accuracies than BayesA models for genotypic and phenotypic prediction (Figure 4, Table S3). In contrast, BayesA resulted in higher correlation for RFbin (Figure 3) and all oligogenic simulated traits (Figure 4), independent of the inclusion of pedigree in the genomic prediction and independent of the simulated dominance effect. The superiority of the additive-dominance models was trait dependent and in some cases opposite accuracy patterns were observed, as it is the case for HT and RFbin (Figure 3).

### Genotypic predictive model strength depends on non-additive effects

The prediction of genotypic values is valuable for genetic improvement programs where breeders can capture additive and non-additive effects by cloning selected individuals. Prediction of dominance effects for each locus is also critical to optimize crossing designs, such as in mate-pair allocation. The inclusion of the Kd kernels matrix in RKHS was equal or worse than the Ka-only kernels for both real traits (Figure 3) and simulated traits (Figure 4). On the other hand, the inclusion of dominance effects in the BayesA model resulted in a better prediction for HT (Figure 3). For simulated traits, the additive-dominance-BayesA showed considerably higher genotypic (Figure 4) and phenotypic (Table S3) prediction accuracy compared to the additive-BayesA in traits with high dominance.

### BayesA models generated higher accuracies in breeding value prediction

The breeding value of one individual represents the component of its genotypic value that is directly transmitted to the progeny. Thus, the breeding value is a critical parameter in the selection of individuals to be used extensively in mating with other individuals in the population. With BayesA and pedigree-based models, it was possible to predict directly the breeding values. The RKHS models, however, generate a prediction of the whole genotypic values, which cannot be split in breeding values, dominance deviation and epistasis. In the simulated the traits, we still reported the correlation between predicted genotypic values and parametric breeding values to assess the accuracy in breeding value prediction. The traditional additive-BayesA and additive-dominance-BayesA based only in marker information estimated the higher accuracy for breeding value prediction for all simulated traits (Figure 4), while pedigree-based models showed the worst accuracies (Table 2).

### Variance components and heritability

One of the most important tasks for a breeder is to make decisions regarding the breeding strategy. Several genetic parameters are evaluated to determine breeding strategies, including the estimation of variance components and the proportion of the genetic variance relative to the phenotypic variance. Here these parameters were estimated using genetic markers, pedigree information or both, for the real and simulated traits. For simulated traits, the parametric values are known, which allowed us to evaluate the capacity of the methods and models to accurately estimate them.

Considering the parametric values of *h^2^* and *d^2^* in the simulated population, the BayesA models that used only genetic markers resulted in the least biased estimate of the genetic parameters (Table S4). The inclusion of pedigree information in BayesA models increased the estimates of heritabilities, and in most cases these parameters were overestimated. Models based only on pedigree also overestimated these genetics parameters: the additive-only pedigree model over estimated *h^2^*, and the additive-dominance pedigree models estimated high *d^2^* for traits with no dominance.

The RKHS based models predict the whole genotypic values. Consequently, estimation of both *h^2^* and *d^2^* is not possible. Nonetheless, *H^2^* can be obtained. The *H^2^* was calculated for the simulated traits and showed that all RKHS based models, regardless of the pedigree inclusion, overestimated this parameter substantially (on average by ∼106%, Table S4). Overestimation is higher in models that included the Kd kernel (RKHS Ka-Kd). Table S5 reports the results of heritabilities estimated with all models for real traits. Similarly, to the simulated data, the estimation of *h^2^* and *d^2^* increased with the pedigree information, and the RKHS based models resulted in much larger estimates of *H^2^*.

### Prediction bias

The regression coefficient (slope) of observed values *vs.* predicted values was used to measure model bias – a slope of one indicates the absence of any bias. The linear regression of simulated data included parametric genotypic values and prediction. For the real data, where the parametric values are unknown, we calculated the slope using phenotype values. The predictions using real data (Table S6) resulted in regression coefficients near one. Most predictions using simulated data resulted in a slope similar to one, with the exception of dominance deviation predictions. In this case, the slopes showed a biased dominance prediction. More specifically, the slope for dominance deviation prediction was less than one in the marker-only models with additive-dominance effects, indicating an underestimation of dominance effects. In contrast, when the pedigree information was considered, the slopes were higher than one, suggesting that the pedigree information contributed to an overestimation of dominance effects (Table S7).

## Discussion

We tested the performance of genomic- and pedigree-based models, with and without non-additive effects, for the prediction of genetic values in complex traits. We used real data from a standard pine tree breeding program in its third generation. Pine traits used for model testing included plant height (HT), diameter at breast height (DBH), and the rust resistance measures RFbin and RFgall, whose narrow sense heritability, prior to least square mean adjustment, were previous reported as 0.31, 0.31, 0.21 and 0.12, respectively (Resende *et al.* 2012b). These traits have different genetic architecture; DBH and HT are polygenic whereas rust resistance is thought to be an oligogenic trait (Resende *et al.* 2012b; Quesada *et al.* 2014). Moreover, HT has significant non-additive effects (Muñoz *et al.* 2014). To expand and validate our conclusions, we simulated six traits with distinct genetic architectures (polygenic and oligogenic) and three dominance levels.

### Pedigree information in genomic predictions

Pedigree and marker information were used separately and in combination, to predict genetic values. In the prediction of breeding and whole genotypic values, the genomic selection model and the combined model (genetic markers + pedigree) were substantially better than the models accounting only for pedigree information. However, the combined model did not improve the prediction accuracies. Previous studies suggest that the use of pedigree data only results in an improvement in prediction when a low marker density is used, as seen in simulated studies (Calus and Veerkamp 2007), as well as in wheat (Crossa *et al.* 2010) and dairy cattle (Vazquez *et al.* 2010). In contrast, pedigree information did not improve model predictions when a higher density of genetic markers were used (Calus and Veerkamp 2007; de los Campos *et al.* 2009b; Vazquez *et al.* 2010). An exception was reported in maize, when prediction models generated with a high-density SNP panel from genotyping-by-sequencing (GBS) was improved with the addition of pedigree data (Crossa *et al.* 2013). A possible explanation for this outcome rests in the fact that the GBS data contains a high frequency of missing data (Crossa *et al.* 2013), resulting in incomplete genomic information.

### Semi-parametric kernel choice

Different kernels can be used to improve the predictions of complex traits in semi-parametric RKHS models. In the current study, the genomic predictions of RKHS Ka-Kd models and the simpler RKHS Ka models yielded similar results. These findings are in agreement with those previously reported by Morota *et al.* (2014) for dairy cattle. These authors did not find increased accuracies with the inclusion of extra kernels in the RKHS Ka. Other kernel comparisons in RKHS also showed that RKHS Ka is a robust choice for the prediction of additive and non-additive effects (Morota *et al.* 2013; Tusell *et al.* 2014).

### BayesA resulted in the highest breeding values accuracy

The BayesA models based only on genetic marker information resulted in the highest accuracies of breeding values prediction in all the simulated scenarios, regardless of the dominance effects. The additive-BayesA and additive-dominance-BayesA models also resulted in similar accuracies for breeding values in additive-dominance traits, despite the fact that the breeding value is also function of dominance effects (Falconer and Mackay 1996). While this result was unexpected, one possible reason for this is the overall low accuracy in the prediction of dominance. The results were also similar to another simulation study that showed higher genetic gain with the additive model compared to an additive-dominance model, even in presence of higher dominance effects (Denis and Bouvet 2012). Furthermore, Nishio and Satoh (2014) also showed that dominance inclusion did not provide higher accuracies in breeding value prediction, even in traits with dominance effects.

### Prediction of whole-genotypic values of traits with distinct genetic architecture

Models with built-in assumptions that some loci have major effects, such as BayesA, usually provide better genomic predictions for simulated oligogenic traits (de los Campos *et al.* 2013), and also for real traits controlled by few genes, such as fat percentage in milk (Habier *et al.* 2011). For genotypic prediction in this study, BayesA generated better predictions than RKHS for RFbin and simulated oligogenic traits. This finding is in agreement with other studies that suggest that rust resistance is an oligogenic trait (Resende *et al.* 2012b; Quesada *et al.* 2014). In the case of simulated additive-dominance oligogenic traits, the additive-dominant-BayesA model resulted in the best predictions of genotypic values.

Our analyses of polygenic simulated traits showed that, for additive-dominant polygenic traits, the RKHS models were better than the additive-BayesA for whole genotypic predictions. These findings agree with those of other authors who argue that RKHS addresses non-additive variation in a non-explicit manner (Gianola *et al.* 2006; Gianola and van Kaam 2008; Morota and Gianola 2014). In addition, RKHS was modestly more accurate when compared to the additive-dominant-BayesA, confirming that RKHS can be explored for predictions in polygenic traits.

In CCLONES population, the RKHS models was slightly more accurate than additive- and additive-dominant-BayesA for HT and DBH. In addition, the additive-dominant-BayesA model was slightly more accurate than the additive-BayesA for HT. This result suggests the presence of non-additive effects in *P. taeda* tree height, which was also previously suggested by Muñoz *et al.* (2014). Similar results were also observed in a genomic selection study by Bouvet *et al.* (2016) which reported higher prediction accuracy of HT in Eucalyptus using models with non-additive effects. In contrast, El-dien *et al.* (2016), did not achieved prediction accuracy increase with inclusion of non-additive effects in HT in an open-pollinated white Spruce population. Altogether, and as expected, we conclude that the increase of prediction accuracy with inclusion of dominance depend of the population and trait.

Similar to Xavier *et al.* (2016), we also fit a model that combined BayesA and RKHS in a single model. This combined model resulted in lower accuracies than additive BayesA for RFbin and simulated oligogenic traits with d^2^ = 0 and d^2^ = 0.1. In addition, the BayesA+RKHS combined model provides lower accuracy than RKHS for HT, DBH and polygenic additive-dominance traits (Table S8). However, the BayesA+RKHS model was slightly more accurate than BayesA for genotypic prediction in oligogenic traits with high dominance (d^2^ = 0.2) and more accurate than RKHS in additive polygenic traits for breeding and genotypic values prediction (Tables S9). In soybean, Xavier *et al.* (2016) reported that BayesA+RKHS and BayesB+RKHS were the most accurate genomic prediction models. These authors suggested that this model could be capturing the breeding values through BayesA and the remaining genetic components with RKHS. In our dataset, this advantage of the combined model was not clear, but further evaluation of this model could be made.

### Heritabilities estimation

When using RKHS based models and pedigree information, the parameters *h^2^*, *d^2^* and *H^2^* were often overestimated. Similarly, others genomic prediction studies in forest species also suggested that pedigree based models generate large and unrealistic estimates of h^2^, when compared with GBLUP (Bouvet *et al.* 2016; El-dien *et al.* 2016).

In quantitative genetics theory, the additive and non-additive effects are typically assumed to be orthogonal and can be divided as independent components from the whole genotypic variance (Falconer and Mackay 1996; Hallauer *et al.* 2010; de los Campos *et al.* 2015). However, in practical situations (absence of Hardy-Weinberg equilibrium, non-random matting), these presuppositions do not hold, and additive and dominance effects are related to each other and not orthogonal. The inability to partition the components can lead to bias estimates of the variance components, affecting the estimates of selection gain as well as the interpretation of the trait architecture. Muñoz *et al.* (2014) indicated that this bias was stronger in pedigree models and recommended the use of genetic markers to partition the additive and non-additive components.

The marker specific variances and therefore the variance component estimates can be influenced by the effect of the priors in Bayesian models (Gianola *et al.* 2009). Nonetheless, in our study, the BayesA models using only markers, generated more reasonable variance components estimates, compared to the parametric values. The inclusion of dominance effects in BayesA resulted in less biased heritabilities estimation for additive-dominance traits, and the traditional additive-BayesA were less biased for *h^2^* estimation in additive traits. This suggests that additive-dominant models should be used for estimation of heritability in cases where the inclusion of dominance effects increase the prediction accuracy. Based on this observation, the estimates for tree height of *h^2^* and *d^2^* using the additive-dominant model equal 0.37 and 0.17 respectively. For DBH, RFbin and RFgall, the additive model is suggested and theirs respective *h^2^* are estimated in 0.52, 0.39 and 0.29 (Table S13). The *h^2^* estimated for HT in this report were similar to the value found in Resende *et al.* (2012b), but higher than reported in Resende *et al.* (2012a). The estimates of *d^2^* for HT were similar to previous report (Muñoz *et al.* 2014), which used the same population but different methods. For DBH, and rust resistance, the *h^2^* obtained in this study, were higher than other authors (Resende *et al.* 2012a, 2012b). Calus and Veerkamp (2007) reported that the inclusion of polygenic effects resulted in better estimates of variance components when compared with models that included only markers - this result that was not replicated in our study.

### Prediction accuracy is affected by the choice of training population

This study contrasted three schemes of training population (TP). The results showed that across families cross validation resulted the lowest accuracy, whereas the setup to within families cross validation produced the best results in most part of cases, being slightly superior than random sample individuals for TP. These results were expected, and further emphasize the importance of genetic relationship among individuals used to fit the model and target individuals for genomic prediction. Genomic Selection models have been shown to capture not only linkage disequilibrium between the markers and causal alleles, but also genetic relationship (Habier *et al.* 2007). The scheme based on random sampling has been commonly used, because it represents a balance between within and across families. These results agree with other studies in forest breeding that showed higher accuracies in the prediction within families than across families (Beaulieu *et al.* 2014b, 2014a). In addition, Albrecht *et al.* (2014) reported higher accuracies in the prediction inside genetic groups than across genetics groups. Hence, one of the most important factors when outlining a genomic selection breeding program is the definition of the target population. The TP needs to resemble, as close as possible, the level of relationship of the target population where GS will be applied.

### CONCLUSION

In summary, we conclude that, in the CCLONES pine population and in our simulation studies, genomic selection is effective compared to phenotypic pedigree selection. The inclusion of pedigree information did not improve the prediction accuracies, suggesting that pedigree computation is not required in genomic prediction programs with reasonable SNP panels. This study also supports the findings that the individuals used to compose the training population should be genetically related to the individuals in the target breeding population. Finally, the BayesA models overcame the RKHS-based models for breeding and genotypic prediction for oligogenic traits, while in polygenic traits, BayesA was suitable for breeding value prediction and RKHS for whole-genotypic prediction.
